# Acute esophageal dilatation detected on computed tomography after radiofrequency ablation of an atrial arrhythmia: A case report

**DOI:** 10.1016/j.hrcr.2026.04.014

**Published:** 2026-04-17

**Authors:** Wataru Sasaki, Hitoshi Mori, Kazuhisa Matsumoto, Yoshifumi Ikeda, Ritsushi Kato

**Affiliations:** Department of Cardiology, Saitama Medical University International Medical Center, Hidaka City, Saitama, Japan

**Keywords:** Atrial fibrillation, Radiofrequency ablation, Periesophageal vagal nerve injury, Posterior wall isolation, Esophageal dilatation, Achalasia-like dysfunction

**Key Teaching Points**

• Posterior wall isolation using radiofrequency ablation remains a high-risk procedure for periesophageal injury, even when ablation index targets are within commonly accepted ranges.
• Patient-specific factors such as advanced age, female sex, and low body mass index significantly increase susceptibility to periesophageal vagal nerve injury. These characteristics should be carefully considered when selecting ablation strategies and energy settings.
• Esophageal or periesophageal injury may occur without significant intraluminal esophageal temperature elevation. The absence of temperature rise does not exclude the risk of vagal plexus injury during posterior wall ablation.
• Selective injury to the periesophageal vagal plexus can result in isolated esophagogastric junction dysfunction without gastroparesis. This case highlights that gastrointestinal manifestations after atrial ablation may vary depending on the specific vagal fibers involved.

## Introduction

Radiofrequency (RF) catheter ablation with pulmonary vein isolation (PVI) has long been established as an effective rhythm-control strategy for atrial fibrillation (AF). However, several procedure-related complications have been reported, including cardiac tamponade, pulmonary vein stenosis, and thromboembolic events. Among these, gastrointestinal complications caused by periesophageal vagal nerve injury (PVNI) represent an important but often underrecognized adverse event.^1^ When present, patients may develop prominent symptoms such as nausea, vomiting, anorexia, and bowel dysfunction, substantially impairing their quality of life. Here, we report a rare case of an elderly woman who developed an acute achalasia-like esophageal dysfunction within 24 hours after RF ablation. Chest computed tomography (CT) demonstrated marked esophageal dilatation without evidence of gastric dilatation, suggesting impaired function of the lower esophageal sphincter or esophagogastric junction owing to possible vagal plexus injury.

## Case report

**Figure 1:**
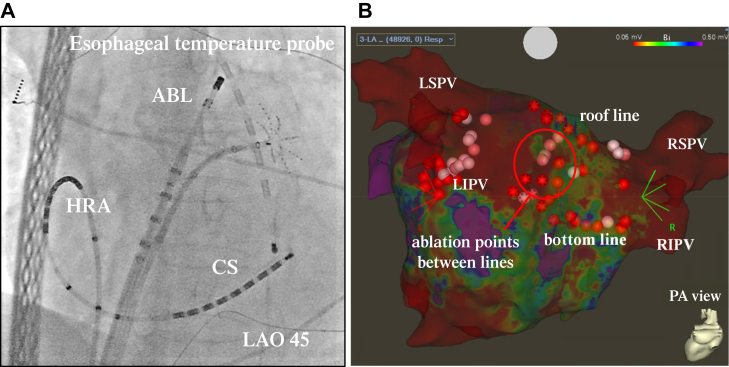
**A:** Catheter positioning and esophageal temperature monitoring during roof line ablation. Fluoroscopic view showing catheter positioning at the initiation point of roof line ablation. The esophagus was located along the left lateral aspect of the left atrium, as indicated by the esophageal temperature probe. A multipolar mapping catheter was positioned within the left atrial appendage. **B:** Left atrial voltage map and ablation sites. The left atrial geometry was created using a multipolar mapping catheter. Ridge-related atrial flutter was terminated by ablation at the left atrial ridge, and substrate-based ablation was performed around the base of the left atrial appendage. Posterior wall isolation was completed by additional ablation targeting residual electrograms within the isolation lines. The *dotted ablation tags* indicate lesions delivered at 90 W for 4 seconds. ABL = ablation catheter; CS = coronary sinus; HRA = high right atrium; LAO = left anterior oblique; LIPV = left inferior pulmonary vein; LSPV = left superior pulmonary vein; PA = posteroanterior; RAO = right anterior oblique; RIPV = right inferior pulmonary vein; RSPV = right superior pulmonary vein.

**Figure 2:**
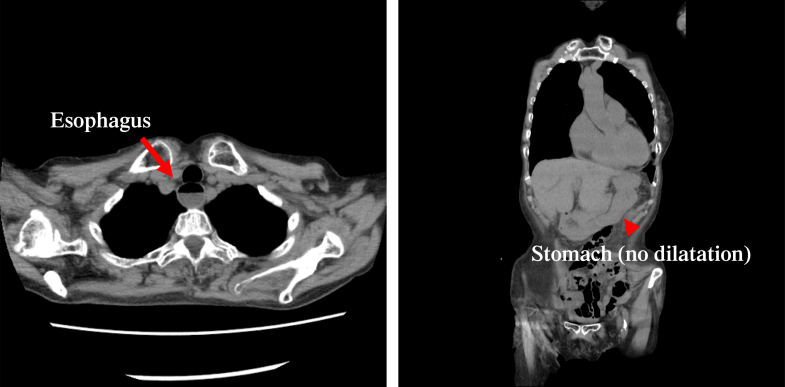
Chest computed tomography findings after ablation. Chest computed tomography images demonstrate marked esophageal dilatation with intraluminal contents, without evidence of gastric dilatation. The red arrows indicate esophageal dilatation at the level of the main bronchus. In contrast, the stomach (indicated by the red arrowhead) shows no evidence of gastric dilatation.

**Supplemental Figure 1:**
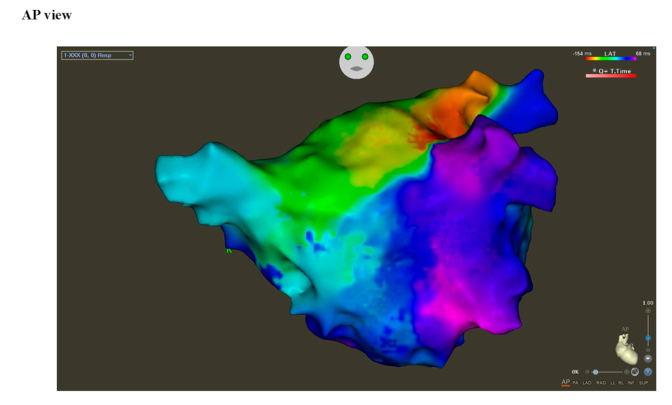


An 85-year-old woman presented with paroxysmal AF accompanied by palpitations. PVI had been performed 5 months earlier using a pulsed-field ablation (PFA) system (VARIPULSE™, Biosense Webster, Irvine, CA). 3 months after that procedure, she experienced recurrent palpitations and was found to have atrial flutter (AFL), for which she was admitted to our hospital for a second ablation session. The procedure was performed using an RF ablation system with 3-dimensional mapping (QDOT, Biosense Webster) under deep sedation. Esophageal temperature monitoring was performed using an EsophaStar™ esophageal temperature probe (Japan Lifeline Co, Ltd, Tokyo, Japan), and fluoroscopic imaging showed that the esophagus coursed along the left lateral aspect of the left atrium (Figure 1A). The previously performed PVI lines showed no recurrence. The clinical AFL was identified as ridge-related AFL (Supplemental Figure 1) and was terminated by an RF application on the left atrial ridge. In addition, several areas of low-voltage substrates were observed around the base of the left atrial appendage; therefore, substrate-based ablation was performed to homogenize these regions. RF applications to these areas were delivered at 50 W with an ablation index target of 450. As a substrate modification strategy, we performed left atrial posterior wall isolation (PWI). RF applications delivered near the esophageal temperature sensor under fluoroscopy were performed at 90 W for 4 seconds, whereas lesions at sites judged to be fluoroscopically remote from the esophagus were applied using 35 W with an ablation index target of 450. When the esophageal temperature exceeded 39°C, energy delivery was discontinued, and ablation was resumed after the temperature had decreased. Because PWI could not be achieved with a single linear lesion set, additional RF applications were delivered to eliminate residual electrograms within the isolation lines, resulting in successful completion of the PWI (Figure 1B). After confirming completion of PWI, substrate modification close to the left atrial appendage was performed based on the electrocardiogram, and the procedure was terminated uneventfully. 5 hours after the procedure, the patient noticed difficulty swallowing, and on the next day, she developed vomiting. Chest CT revealed esophageal dilatation with intraluminal contents, whereas no evidence of gastric dilatation was observed (Figure 2). She was diagnosed as having esophageal dysfunction secondary to PVNI, without evidence of gastric nerve involvement. Oral intake was discontinued, and total parenteral nutrition was initiated. Mosapride citrate hydrate and the traditional Japanese herbal medicine, rikkunshito, were initiated. 1 week after the procedure, an esophagography study demonstrated preserved peristaltic passage of contrast material from the esophagus into the stomach (Supplemental Video). Her diet was gradually advanced from oral nutritional supplements to liquid meals and eventually to regular food, with careful monitoring for recurrence of symptoms. Given that gastrointestinal symptoms did not reappear, she was discharged on postoperative day 25. She remains asymptomatic during outpatient follow-up.

## Discussion

**Figure 3:**
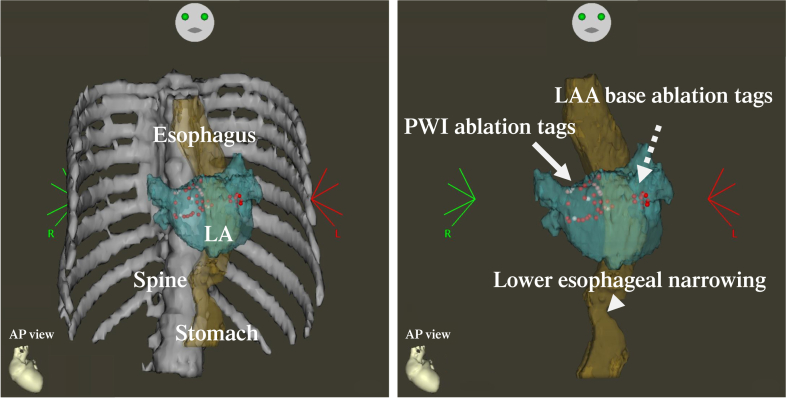
Spatial relationship between the LA, esophagus, spine, and ablation sites. *Left:* Electroanatomic map demonstrating the spatial relationship among the LA, spine, and esophagus. *Right:* Electroanatomic map showing ablation sites in relation to the esophagus. Ablation was delivered along the LA ridge, the base of the LAA (*white dashed arrows*), and the posterior wall (*white arrows*). Notably, lower esophageal narrowing was observed distal to the ablation sites (*white arrowhead*), rather than directly opposite the sites of radiofrequency application, suggesting selective involvement of the periesophageal vagal plexus. AP = anteroposterior; LA = left atrium; LAA = left atrial appendage; PWI = posterior wall isolation.

**Supplemental Figure 2:**
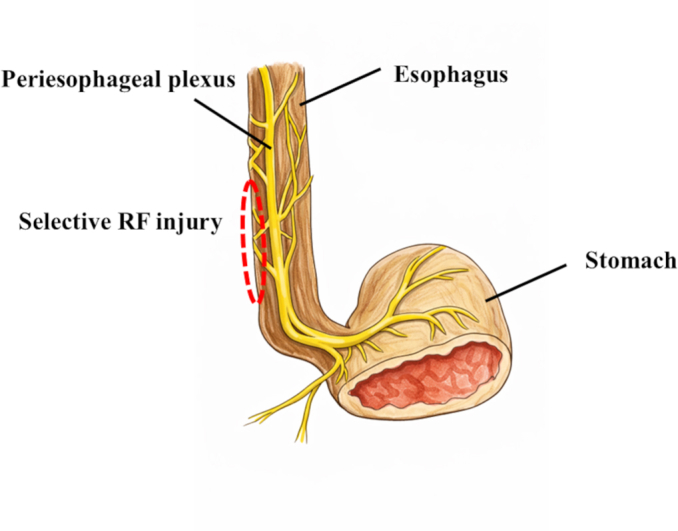


Most reported cases of PVNI after AF ablation have predominantly presented with gastric hypomotility or pyloric dysfunction.^2^^,^^3^ However, PVNI can also involve other components of the gastrointestinal system, and biliary dysfunction such as acalculous cholecystitis and sphincter of Oddi spasm has been reported. Lower gastrointestinal symptoms, including constipation or ileus-like presentations, have also been described, although dedicated reports focusing on colonic dysmotility remain limited.^4^^,^^5^ In contrast, the present case demonstrated marked esophageal dilatation without gastric distension, suggesting a distinct mechanism preferentially affecting the esophagogastric junction. Anatomically, the posterior left atrial wall is separated from the esophagus by only a few millimeters, with the anterior esophageal vagal plexus coursing within this narrow space.^6^ Injury to specific components of this plexus may result in different clinical phenotypes. Although damage to vagal pathways regulating gastric motility leads to gastric dilatation and delayed emptying, selective impairment of fibers mediating lower esophageal sphincter relaxation may cause esophagogastric junction outflow obstruction and an achalasia-like presentation, as observed in this case. The absence of gastric involvement in our patient supports the latter mechanism. In the present case, the previous PVI performed during the initial ablation procedure was complete; therefore, creation of roof and bottom lines anterior to the esophagus was not required for PWI. However, because PWI could not be achieved with standard linear lesions, additional ablation was delivered within the posterior left atrium along the right lateral border of the esophagus (Figure 3), which we consider to be the most likely cause of the subsequent esophageal dilatation. To illustrate the presumed anatomic mechanism underlying the selective esophagogastric junction dysfunction observed in this case, a schematic illustration of the periesophageal vagal plexus based on previous anatomic studies is presented in Supplemental Figure 2. Therefore, the resulting pattern of esophageal dilatation without gastric dilatation is not inconsistent with selective injury to vagal plexus fibers running along the right margin of the esophagus. Furthermore, interindividual variability in the esophageal position and vagal nerve branching patterns, as well as esophageal migration during ablation,^7^ may explain why similar ablation strategies result in different gastrointestinal manifestations. Although secondary achalasia after PVI has been previously reported,^8^ to the best of our knowledge, this is the first case demonstrating isolated esophageal dilatation without gastric distension on CT. Several risk factors for PVNI have been proposed, including older age, female sex, and low body mass index.^9^ These characteristics were all present in our patient, who was an elderly, thin woman, placing her at higher susceptibility for this type of complication. In such high-risk patients, even ablation parameters that are commonly considered acceptable for PWI may increase the likelihood of collateral injury, underscoring the need for careful individualization of energy delivery. In this context, previous studies have suggested that posterior wall ablation using standard or even reduced energy settings does not completely eliminate the risk of esophageal injury, indicating that patient-specific vulnerability plays a critical role beyond procedural parameters alone.^10^ Moreover, because the periesophageal vagal plexus extends beyond the area directly monitored by an esophageal temperature probe, careful consideration of ablation settings is warranted in regions adjacent to the full transverse width of the esophagus as assessed by preprocedural imaging, even in the absence of significant temperature elevation. In recent years, PFA has gained widespread attention as a nonthermal ablation modality that induces myocardial apoptosis by electroporation rather than resistive heating. PFA seems to significantly reduce the risk of collateral thermal injury to adjacent structures, and reports of vagal nerve injury after PFA are exceedingly rare.^11^ Moreover, there have been reports demonstrating the safety and efficacy of PWI using PFA.^12^ In patients with multiple risk factors for PVNI who are scheduled to undergo PWI, consideration of nonthermal energy sources such as PFA may be warranted to enhance procedural safety.

## Conclusion

We report a rare case of acute esophageal dilatation without gastric distension after RF catheter ablation, likely caused by PVNI predominantly affecting the esophagogastric junction. This case highlights the possibility that vagal nerve injury after PWI may present as isolated esophageal dysfunction rather than typical gastric hypomotility. In patients with multiple susceptibility factors, careful procedural planning and consideration of nonthermal ablation strategies may be important to enhance procedural safety.

## Disclosures

H.M. received lecture fees from Biosense Webster Japan and Boston Scientific Japan. All other authors have no conflicts of interest to disclose.

## Footnotes

Appendix Supplementary data associated with this article can be found in the online version at https://doi.org/10.1016/j.hrcr.2026.04.014.
