# Supplementary material for: Acute esophageal dilatation detected on computed tomography after radiofrequency ablation of an atrial arrhythmia: A case report
Source: HeartRhythm Case Rep. 2026 Apr 17;12(7):693–6. doi: 10.1016/j.hrcr.2026.04.014 (PMC13379347; doi:10.1016/j.hrcr.2026.04.014)
Supplement: Supplemental Legend [file mmc1.docx]

**Supplement Figure Legend**

**Supplementary Figure 1.**

Three-dimensional mapping of the clinical A F L, identified as ridge-related AFL.

**Supplementary Figure 2.**

Schematic illustration of the periesophageal vagal plexus based on previous anatomical studies.
