# Supplementary material for: Acute esophageal dilatation detected on computed tomography after radiofrequency ablation of an atrial arrhythmia: A case report
Source: HeartRhythm Case Rep. 2026 Apr 17;12(7):693–6. doi: 10.1016/j.hrcr.2026.04.014 (PMC13379347; doi:10.1016/j.hrcr.2026.04.014)
Supplement: Supplemental Video 1 [file mmc2.docx]

**Supplement Video Legend**

**Supplementary Video 1.**

An esophagography study demonstrated preserved peristaltic passage of contrast material from the esophagus into the stomach
